# Mitochondrial VDAC1: A Key Gatekeeper as Potential Therapeutic Target

**DOI:** 10.3389/fphys.2017.00460

**Published:** 2017-06-30

**Authors:** Amadou K. S. Camara, YiFan Zhou, Po-Chao Wen, Emad Tajkhorshid, Wai-Meng Kwok

**Affiliations:** ^1^Department of Anesthesiology, Medical College of WisconsinMilwaukee, WI, United States; ^2^Cardiovascular Center, Medical College of WisconsinMilwaukee, WI, United States; ^3^Department of Assay Development, HD BiosciencesShanghai, China; ^4^Department of Biochemistry, Beckman Institute for Advanced Science and Technology, Center for Biophysics and Quantitative Biology, University of Illinois at Urbana-ChampaignUrbana, IL, United States; ^5^Department of Pharmacology and Toxicology, Medical College of WisconsinMilwaukee, WI, United States

**Keywords:** mitochondria, cardiac ischemia/reperfusion, Alzheimer's disease, neoplastic diseases, molecular dynamics, voltage dependent anion channel, hexokinase, post-translational modification

## Abstract

Mitochondria are the key source of ATP that fuels cellular functions, and they are also central in cellular signaling, cell division and apoptosis. Dysfunction of mitochondria has been implicated in a wide range of diseases, including neurodegenerative and cardiac diseases, and various types of cancer. One of the key proteins that regulate mitochondrial function is the voltage-dependent anion channel 1 (VDAC1), the most abundant protein on the outer membrane of mitochondria. VDAC1 is the gatekeeper for the passages of metabolites, nucleotides, and ions; it plays a crucial role in regulating apoptosis due to its interaction with apoptotic and anti-apoptotic proteins, namely members of the Bcl-2 family of proteins and hexokinase. Therefore, regulation of VDAC1 is crucial not only for metabolic functions of mitochondria, but also for cell survival. In fact, multiple lines of evidence have confirmed the involvement of VDAC1 in several diseases. Consequently, modulation or dysregulation of VDAC1 function can potentially attenuate or exacerbate pathophysiological conditions. Understanding the role of VDAC1 in health and disease could lead to selective protection of cells in different tissues and diverse diseases. The purpose of this review is to discuss the role of VDAC1 in the pathogenesis of diseases and as a potentially effective target for therapeutic management of various pathologies.

## Introduction

Mitochondria are vital for cellular metabolism, and are the primary source of ATP generated via oxidative phosphorylation. In addition to their role as the cellular powerhouse, they are key organelles that are intimately involved in a myriad of complex signaling cascades that regulate cell survival and death. Consequently, mitochondrial dysfunction has been implicated in the etiology of numerous human maladies, including cardiovascular, neurodegenerative, and neoplastic diseases (Camara et al., 2010). Thus, alleviating or preventing mitochondrial dysfunction will contribute to mitigating the severity or progression of the development of diseases.

**Figure 1:**
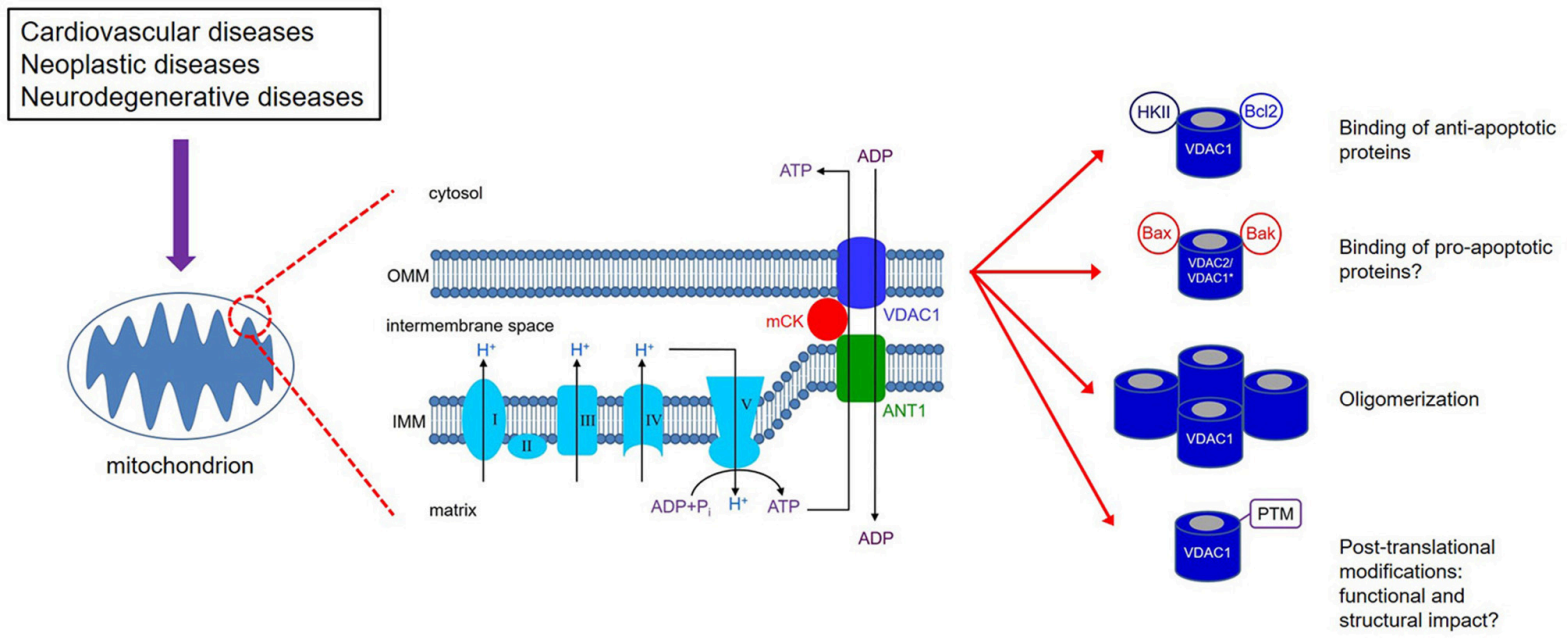
VDAC1 is expressed on the outer mitochondrial membrane (OMM). Together with ANT1 on the inner mitochondrial membrane (IMM) and mitochondrial creatine kinase (mCK), the VDAC1-ANT1-mCK complex regulates the exchange of ATP and ADP between the mitochondria and cytosol. VDAC1 functions as a receptor for anti- and pro-apoptotic proteins and, consequently, contributes to cell survival and cell death. ^*^Bax/Bak binding to VDAC1 has not been definitively shown as various studies report on conflicting results; on the other hand, Bax/Bak binding to VDAC2 has been supported by more consistent results. VDAC1 oligomerization can result in increased permeability of the OMM; however, the mechanism that leads to apoptosis has not been clearly defined. Various types of post-translational modificantions (PTMs) of VDAC1 have been reported, although their impact on channel function and subsequently on mitochondrial function is not well understood.

The elaborate structure of a mitochondrion is important for the normal and efficient functioning of the organelle. Mitochondria have two membranes, an outer mitochondrial membrane (OMM) and an inner mitochondrial membrane (IMM), separated by the inter-membrane space (IMS) (Figure 1). The electron transport chain (ETC) complexes (complexes I-IV) are localized on the IMM, while the OMM separates the mitochondrion from the cytosolic environment. The main conduit through which metabolites and nucleotides traverse the OMM is the voltage-dependent anion channel (VDAC), also known as mitochondrial porin (Mihara and Sato, 1985; Kleene et al., 1987). The VDAC is activated during depolarizing potentials and remains in an open state in the voltage range of approximately −40 to +40 mV (Rostovtseva and Colombini, 1996, 1997; Hodge and Colombini, 1997). In addition, as its name implies, it shows both ion selectivity and voltage dependence. In the open (high conductance) state, the channel is permeable to organic anions, including respiratory substrates, ATP, ADP, Pi, and to metabolites (Rostovtseva and Colombini, 1996; Hodge and Colombini, 1997). In the closed (low-conductance) state, the channel transports cations, e.g., K^+^, Na^+^ and Ca^2+^. Under physiological conditions, VDAC function is modulated by tubulin, a key component of the cytoskeleton (Rostovtseva et al., 2008), which would limit mitochondrial metabolism and thereby alter the IMM potential (ΔΨ_m_) (Rostovtseva et al., 2008; Rostovtseva and Bezrukov, 2012). VDAC function is also associated with NADH oxidation and thus plays a role in cellular redox mechanisms (Komarov et al., 2005). Furthermore, it also plays a key role in mitochondrial-mediated apoptotic signaling due to its ability to interact with members of the pro- and anti-apoptotic family of proteins, including the Bcl-2 family of proteins and cytosolic kinases (Figure 1), e.g., hexokinase (HK), involved in intermediary metabolism (Doran and Halestrap, 2000; Shimizu et al., 2001; Pastorino et al., 2002). Because of its involvement in regulating mitochondrial function and in serving as a gatekeeper for cell death and survival signaling pathways, VDAC can potentially be an attractive and effective therapeutic target in the management of various human diseases.

The main premise of this review is that alterations of VDAC structure and function contribute to pathological states, either directly or indirectly. It is evident that knowledge of the role of VDAC in mitochondrial function in normal and pathological conditions is crucial not only for our understanding of the basic cause of mitochondrial related diseases, but also for developing therapeutic strategies for mitigating a given disease. Here we present an overview of the physiological role of VDAC, particularly voltage-dependent anion channel 1 (VDAC1), and the impact of post-translational modification (PTM) triggered by pathophysiological stresses in the progression of diseases. Specific examples of VDAC1 role in neoplastic and neurodegenerative diseases, and cardiac ischemia and reperfusion (IR) injury are presented. In addition, the review will also summarize the recently reported findings of VDAC structure and function at the molecular level. Lastly, the review will also address the controversial existence of VDAC in the plasma membrane identified in some tissue types and its physiological implications. A perspective of the roles of VDAC1 in health and disease and the potential implications in therapy is also noted.

## Overview of VDACs

The VDACs are the most abundant protein in the OMM with a molecular weight of approximately 32 kD. Under normal physiological conditions, VDACs function in tandem with the IMM adenine nucleotide translocase (ANT), via the mitochondrial creatine kinase (mCK) in the IMS (Figure 1; Schlattner et al., 2006; Guzun et al., 2012). The conformational states of VDAC are voltage-dependent and exhibit different selectivity and permeability for small ions, showing a preference for anions in the open state and for cations in the closed state (Hodge and Colombini, 1997). Therefore, they are considered the principal sites for the exchange of metabolites and small solutes between the IMS and the cytosol (Camara et al., 2010, 2011). The efficient transfer of energy metabolites across mitochondria depends on the interaction between VDAC, mCK, and ANT, ANT/mCK/VDAC, which is fostered by physiological [Ca^2+^] (Kottke et al., 1988; Saks et al., 2006).

Ca^2+^ released from the sarcoplasmic reticulum (SR) or endoplasmic reticulum (ER) enters mitochondria via the VDAC, which is in close proximity to the SR within the mitochondrial-associated membrane (MAM) domain (Vance, 2014). This inter-organelle communication (SR-mitochondria domain) through MAM is able to coordinate cellular metabolism and preserve OMM integrity (Vance, 2014; Gomez et al., 2016). It is proposed that VDAC1 selectively transfers apoptotic Ca^2+^ signals from SR to mitochondria (De Stefani et al., 2012). Along with other mitochondrial proteins (ANT, PiC, and TSPO), VDACs were thought to be a constituent of the mitochondrial permeability transition pore (mPTP), a mega channel complex involved in cell protection and cell death (Javadov et al., 2009a; Camara et al., 2010, 2011). However, subsequent genetic studies showed mPTP opening in the absence of these proteins, suggesting that they are not an integral component of the mPTP structure, but rather may play regulatory roles in the pore formation (Kokoszka et al., 2004; Krauskopf et al., 2006; Baines et al., 2007). Recent evidence points to the F_0_F_1_-ATP synthase as the major constituent of the mPTP (Giorgio et al., 2013; Carraro et al., 2014). Although the exact mechanisms of the ATP synthase transformation into mPTP are still being actively investigated, a very recent work shows that the catalytic site of the F_0_F_1_-ATP synthase β subunit constitutes the Ca^2+^ trigger site that in turn induces a conformational change and transition of the ATP synthase to a channel (Giorgio et al., 2017). Opening of the mPTP is an IMM event, as it occurs in mitoplasts, i.e., mitochondria stripped of the OMM (Sileikyte et al., 2011). However, VDACs participate in OMM permeabilization that is independent of the mPTP (Doran and Halestrap, 2000) and, as such, they are key in determining the functional integrity of mitochondria and cell fate, i.e., survival or death. VDACs are also considered to play crucial roles in (1) the release of reactive oxygen species (ROS), (2) providing an anchoring site for HK binding, (3) apoptosis-mediated release of cytochrome *c*, and (4) the interaction with the Bcl-2 family of proteins (Shimizu et al., 2000b, 2001; Tsujimoto and Shimizu, 2000; Azoulay-Zohar et al., 2004). For example, blocking VDAC with Konig's polyanion or VDAC antibodies inhibited superoxide-induced release of cytochrome *c* from mitochondria (Madesh and Hajnoczky, 2001). However, VDAC closure can also lead to diminished exchange, and eventual buildup of metabolites resulting in swelling and rupture of the OMM (Vander Heiden et al., 2000). Under conditions of lethal oxidative stress, VDACs can contribute to the pro-apoptotic mitochondrial permeabilization of the OMM either via homo-oligomerization resulting in dimers and/or trimers of VDAC (Yang et al., 2012) or hetero-oligomerization with pro-apoptotic cytosolic proteins (Shimizu et al., 2001). Since VDACs constitute a multiple genetic family with multiple variants, it is reasonable to propose that different functions may be relegated to distinct isoforms. Below we provide additional information on the mPTP and the other means by which mitochondria permeability of the OMM is altered independent of the mPTP.

### VDAC isoforms

To date, three isoforms of the mammalian VDAC have been identified: VDAC1, 2, and 3, of which VDAC1 is the most abundantly expressed. In mammals, three isoforms of VDAC are encoded by three different genes that share some conserved structure (Sampson et al., 1997; Young et al., 2007). The pore-forming voltage-dependent characteristics of VDAC1 and VDAC2 have been amply demonstrated, but that of VDAC3 has only been recently examined in detailed biophysical and electrophysiological studies (Checchetto et al., 2014; Okazaki et al., 2015). VDAC1 and VDAC2 are thought to be co-localized within the same restricted area in the OMM, while VDAC 3 is broadly distributed on the OMM (Neumann et al., 2010; Okazaki et al., 2015). Both VDAC1 and VDAC2 display similar ion selectivity and the unique characteristic voltage-dependence. That is, channel conductance is greatest at membrane voltages near 0 mV, within a range of −40 to +40 mV. At these voltages, VDAC1 and VDAC2 function predominantly as anion channels, while at voltages outside of this range they function as cation channels, permeable to ions, such as Ca^2+^. In contrast, a recent study by Okazaki et al. showed that channel gating of VDAC3 did not exhibit the typical voltage gating (Okazaki et al., 2015), and Checchetto et al. (2014) also showed that the electrophysiological properties of recombinant human VDAC3 are different from VDAC1 and VDAC2.

The specific functions of the VDAC isoforms have not been fully defined. They are believed to be involved in the coupling of cellular energy demand to mitochondrial ATP production insofar as they are the main conduit for transport of metabolites across the OMM (Anflous et al., 2001; Anflous-Pharayra et al., 2011). The different isoforms also have relegated functions. Gene knock out (KO) of VDAC isoforms in mice has shed some light on their respective physiological relevance. It has been suggested that VDAC3 might be evolutionarily distinct, and thus might have other functions different from VDAC1 and VDAC2 (Sampson et al., 1996). VDAC3, whose primary role has been in sperm motility, has been less protective against deleterious ROS when compared to VDAC1 and VDAC2 (De Pinto et al., 2010a). While VDAC1 and VDAC3 KO are viable, VDAC2 gene deletion is embryonically lethal (Baines et al., 2007). Furthermore, this impact of VDAC2 KO on embryonic lethality could not be compensated by overexpression of VDAC1 and VDAC3. In addition, whether VDAC2 could compensate for the deletion of VDAC1 and/or VDAC3 is challenging to ascertain due to the lethality of removal of the *Vdac2* gene (Cheng et al., 2003). Consequently, in the absence of VDAC2, the pro-apoptotic protein Bak is unrestricted in its ability to instigate cell demise, potentially leading to early embryonic death (Cheng et al., 2003; Bernardi et al., 2015). Thus, VDAC2 is considered vital for cell survival. Indeed, it has also been postulated to be cytoprotective by sequestering Bak (Cheng et al., 2003), which if translocated to the OMM, may induce permeabilization.

However, recent observations (discussed later) show that VDAC1 also interacts with Bak in mediating cell death. VDAC1 is thought to control the metabolic crosstalk between mitochondria and the cytosol, by regulating the influx and efflux of metabolites, cations and nucleotides across the OMM for efficient bioenergetics. Consistent with this notion, it has been reported that human VDAC1 deficiency showed compromised pyruvate oxidation and ATP production (Huizing et al., 1996). Later on, Anflous et al. (2001) and Anflous-Pharayra et al. (2011) reported that VDAC1 deficiency caused multiple defects in the ETC complexes in both oxidative and glycolytic striated muscle biopsies. Structural aberration of mitochondria was also observed. With impaired oxidative phosphorylation and reduced ATP levels, the cells cannot maintain structural and functional integrity. This includes loss of ion homeostasis resulting in irreparable cell injury and concomitantly cell death, primarily through necrosis. In contrast, VDAC3 null showed limited impairment in muscle metabolism.

A key factor in mitochondrial bioenergetics regulation is the association of mCK with VDAC1 (Schlattner et al., 2006; Guzun et al., 2012; Lemeshko, 2016), which allows for the efficient exchange of ADP and ATP between the matrix and the cytosol that ultimately regulates mitochondrial respiration (Figure 1). It is not known whether similar association exists between VDAC2 and mCK. However, VDAC1 and VDAC2 deficient mouse embryonic stem cells, but not VDAC3 deficient cells, displayed reduced cytochrome c oxidase (complex IV) activity (Wu et al., 1999) which portends diminished ADP/ATP shuttle across the OMM. The preponderance of the evidence reported to date indicates that the *in vivo* roles of these isoforms of VDAC may fulfill different functions; but identifying their specific physiology is encumbered by limitations in the current approach of assessing their different roles.

### VDAC structure

**Figure 2:**
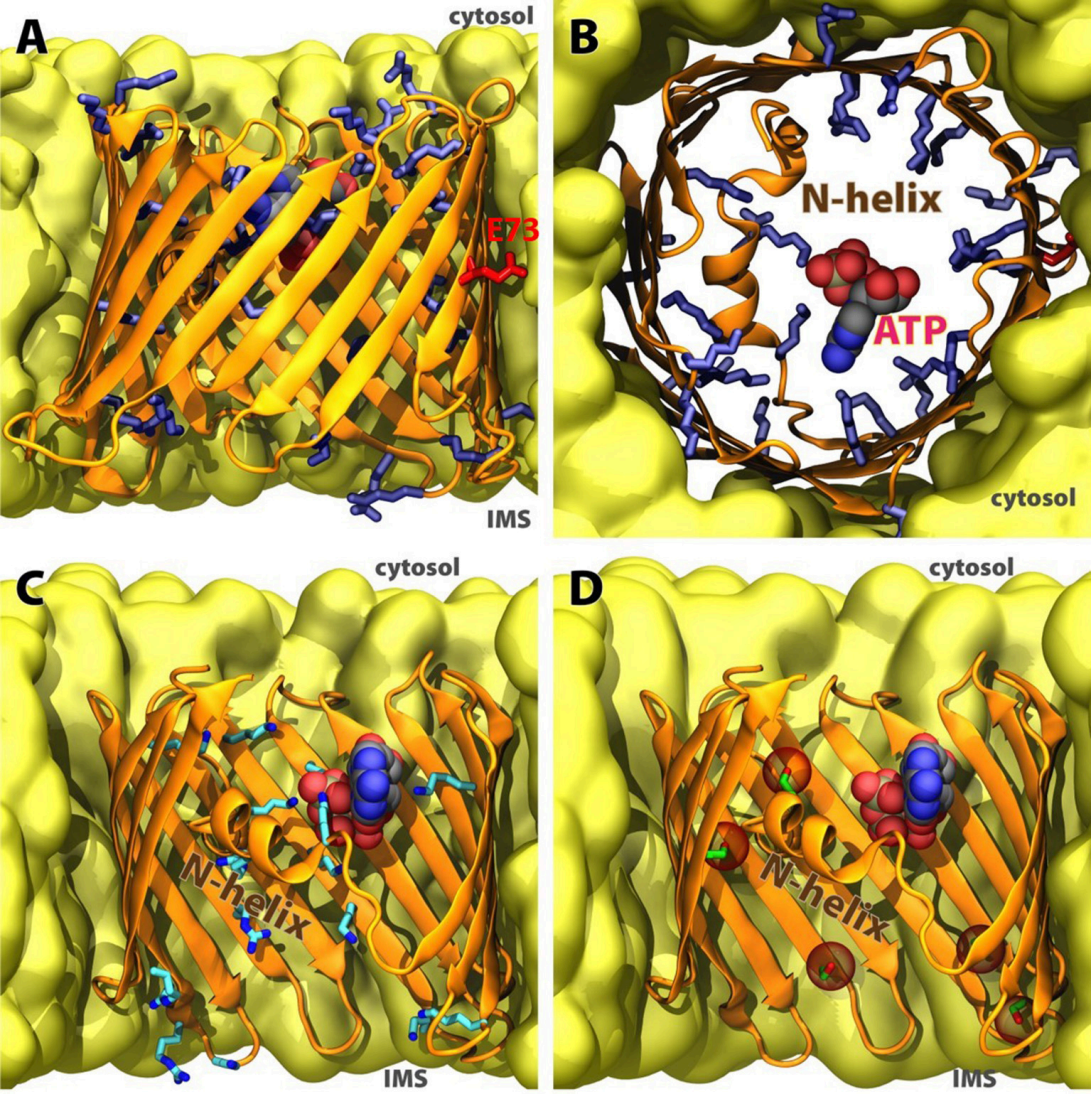
Structure of human VDAC1 in membrane. **(A)** Sideview and **(B)** cytoplasmic topview of the beta-barrel, with basic residues in blue sticks, acidic E73 in red sticks, and ATP in spheres. **(C)** Significant ATP-interacting residues along permeation pathway (data adopted from Choudhary et al., 2014). **(D)** Currently confirmed phosphorylation sites in human VDAC1 (data adopted from Martel et al., 2014), positions of phospho-serines and phospho-threonine highlighted in red spheres. C-terminal part of the beta-barrel is omitted in **(C,D)** for clarity.

Recent studies reported on a high-resolution crystal structure of VDAC1 (Figure 2; Bayrhuber et al., 2008; Hiller et al., 2008; Ujwal et al., 2008). The conventional notion of the channel/protein is a β-barrel trans-membrane pore (hydrophilic) with 19-stranded β-sheets. The N-terminal segment consists of an α-helix oriented toward the interior wall of the pore (Bayrhuber et al., 2008; Erbse et al., 2008), and is thought to act as the voltage sensor that leads to changes in conductance and ion selectivity (Blachly-Dyson et al., 1990; Thomas et al., 1993). However, the underlying mechanism is not well understood. The N-terminal of VDAC1 is crucial for channel gating and its ablation leads to loss of voltage gating (Koppel et al., 1998; Abu-Hamad et al., 2009). The mechanisms for the open-close transition of the channel by translocation of the N-terminal region are still not clear. It has been postulated that the N-terminal gates VDAC1 by moving in and out of the pore (Geula et al., 2012). However, electrophysiological and structural studies and molecular dynamics simulations implicate the N-terminus with less mobility that serves as a stabilizing factor in a non-rigid pore (Bayrhuber et al., 2008; Hiller et al., 2008; Schneider et al., 2010; Zachariae et al., 2012). Removal of the N-terminus resulted in a partial collapse of the pore resulting in an elliptically-shaped conduction pathway (Zachariae et al., 2012). In this scenario, the N-terminus stabilizes VDAC1 in the open conformation, while a closed conformation is associated with partially collapsed wall of the pore, resulting in decreased conductance and a change in selectivity from anions to cations. The N-terminal is also thought to be important for the regulation of metabolite fluxes through the channel; it mediates protein-protein interactions and the oligomerization of VDAC1 with pro- or anti-apoptotic proteins, HK and Bcl2 (Figure 1), respectively. This suggests external orientation of the N-terminal in the pore (Shoshan-Barmatz et al., 2014). The N-terminal is also thought to be capable of partially narrowing the pore, and consequently is a crucial component of the channel's gating mechanism.

It is worth noting that the high resolution 3-dimensional crystal structure is not without some controversy (Colombini, 2009). The reported structure does not accommodate some of the structural constraints predicted from biochemical and biophysical studies. From those studies, a VDAC structure with 13, rather than 19, β-strands and a single α-helix was envisioned. The argument against the 19 β-strand model was based largely on the validity of the use of refolded proteins in the structural studies that utilized NMR and X-ray diffraction techniques. On the other hand, the case against the 13 β-strand + 1 α-helix hybrid model was an energetically unstable model. It should also be noted that a large body of the biochemical and biophysical studies were conducted using reconstituted VDAC in planar lipid bilayers. Consequently, experimental constraints exist on both sides of the argument, and future studies will be needed to provide a more definitive structure of VDAC in a “native” environment. Nevertheless, novel insights into VDAC structure and function relationships (Figure 2) have been provided by Molecular Dynamic (MD) simulations as discussed below.

### Binding of pro- and anti-apoptotic proteins

As a major gateway in and out of mitochondria, VDAC mediates an intimate dichotomy between metabolism and cell death (Maldonado et al., 2010; Camara et al., 2011; Maldonado and Lemasters, 2012). The delicate balance in the interactions between pro- (e.g., Bcl family of proteins) and anti-apoptotic [e.g., hexokinases (HK), I and II] proteins with VDAC (Figure 1) is central in this dichotomy. These interactions alternatively promote or prevent cell injury by apoptosis/necrosis (Shimizu et al., 2001; Leanza et al., 2014). For example, VDAC1 can act as a receptor on the cytosolic side for HK to bind and, this way, has easy access to mitochondrial ATP for its catalytic activity (Schlattner et al., 2006). HK catalyzes the conversion of glucose to substrates critical for oxidative phosphorylation (Arora and Pedersen, 1988). Of the two HK isoforms, HK II is over-expressed in many types of cancer cells, and its interaction with VDAC1 contributes to their unrestricted growth (discussed below). VDAC1 also interacts with several members of the Bcl-2 family of proteins that are key regulators of apoptosis. Although the precise mechanisms by which the Bcl-2 family of proteins regulate apoptosis is not well established, VDAC1 has been shown to be a target for both the anti-apoptotic (Bcl2 and Bcl-xL) and pro-apoptotic (Bax, Bak, and Bim) proteins (Shimizu et al., 2000c, 2001; Vander Heiden et al., 2001; Pastorino et al., 2002; Arbel et al., 2012; Huang et al., 2013; Liu et al., 2015). Interaction of the pro-apoptotic Bcl-2 members with VDAC1 is thought to lead to OMM permeabilization that is likely associated with the release of cytochrome *c* from the IMS. On the other hand, interaction with the anti-apoptotic proteins inhibits VDAC1 oligomerization to prevent OMM permeabilization and cell damage. The aforementioned discussions strongly support the role of VDAC in maintaining normal mitochondria function. The interaction of VDAC with other cytosolic proteins, i.e., pro- or anti-apoptotic, for cell survival or death provides a potential therapeutic target to mitigate cell injury, as in IR injury and neurodegenerative diseases, or instigate cell death, as in neoplastic diseases (discussed below).

### VDAC and mitochondrial membrane permeabilization

The role of VDAC in mitochondrial membrane permeabilization has been mired in controversy, particularly with the report that VDAC isoforms are dispensable for opening of the mPTP. Previous electrophysiological and biochemical studies have provided evidence of a molecular model of mPTP that consisted of VDAC on the OMM, ANT on the IMM, and cyclophilin D in the matrix (Szabo and Zoratti, 1993; Szabo et al., 1993; Crompton et al., 1998; Zheng et al., 2004). However, other studies have contradicted those findings, with reports that closed conformation of VDAC increases Ca^2+^ permeability, and thus should accelerate opening of mPTP (Tan and Colombini, 2007; Tikunov et al., 2010). A more compelling study that questions VDAC as a component of mPTP came from genetic studies, where knockout of all three VDAC isoforms did not abolish opening of mPTP, though cells became more susceptible to death stimuli (Baines et al., 2007). Yet, results from genetic studies are not necessarily definitive. Knockdown, or for that matter overexpression, of proteins can be associated with physiological adaptations. In the case of VDAC knockdown, it is conceivable that alternative pathways allow for the exchange of metabolites across the OMM, indicative of the ability of mitochondria to compensate for the loss of VDAC. Recent reports continue to provide evidence of the involvement of VDAC1 in contributing to mPTP opening. For example, overexpression of microRNA-7, a small non-coding RNA, was reported to prevent opening of mPTP by downregulating VDAC1 (Chaudhuri et al., 2016). In an investigation of the role of mitochondrial fission factor in cardiac microvascular IR injury, preventing the oligomerization of VDAC1 and detachment of HKII resulted in the inhibition of mPTP opening (Zhou et al., 2017). In addition to VDAC, ANT and the phosphate carrier (PiC) have been proposed to be part of mPTP (De Macedo et al., 1993; Brustovetsky and Klingenberg, 1996; Bauer et al., 1999; Haworth and Hunter, 2000; Leung et al., 2008; Varanyuwatana and Halestrap, 2012), but studies utilizing genetic knockdown of the PiC or ANT do not support those observations (Kokoszka et al., 2004; Gutierrez-Aguilar et al., 2014). In fact, the molecular identity of mPTP remains unresolved, although multiple lines of evidence support the role of cyclophilin D as a key regulator of the pore (Javadov and Kuznetsov, 2013). A more recent molecular candidate include the transformation of the F_0_F_1_-ATPase to mPTP (Bernardi et al., 2015). Based on these confounding results, it is conceivable that mPTP may be comprised of multiple molecular entities (Bonora et al., 2013; Giorgio et al., 2013; Alavian et al., 2014; Carraro et al., 2014).

The complexity (or uncertainty) of the role of VDAC1 in contributing to mPTP opening is likely due to strong evidence that it is involved in the permeabilization of the OMM, termed mitochondrial outer membrane permeabilization (MOMP). As mentioned above, some studies have demonstrated that Bax binds to VDAC1; this association apparently results in the formation of a channel complex with sufficient pore size capable of releasing cytochrome *c* from the IMS to the cytosol and trigger caspase 9 initiated apoptosis (Shimizu et al., 2000a). However, the interaction of VDAC1 with Bax is not without controversy, as this association has been disputed from planar lipid bilayer electrophysiological studies (Rostovtseva et al., 2004). Discrepant results can, in part, be due to experimental conditions. Recently, VDAC1-Bax interactions was observed in cultured neurons that were dependent on the detergent used in the preparation for immunoprecipitation (Huckabee and Jekabsons, 2011). Specifically, the use of digitonin, but not CHAPS, preserved the VDAC1-Bax association. Additionally, VDAC1 can also trigger MOMP in the absence of Bax via oligomerization (Huang et al., 2015).

Voltage-dependent anion channel 1 (VDAC1) homo-oligomerization or hetero-oligomerization could result in a “large” pore that allows the release of cytochrome *c* into the cytosol, although the structural details of increased pore size due to oligomerization is not known. Functionally, oligomerization of VDAC1 can lead to OMM permeabilization. Although oligomerization-induced “open channel conformation” is thought to contribute to apoptosis via OMM permeabilization, there is debate as to whether the closed channel conformation also results in apoptosis. The closed, or low conductance, state of VDAC1 has higher selectivity for cations over anions. Thus, greater influx of Ca^2+^ ions into mitochondria is predicted in the closed conformation (Tan and Colombini, 2007). The resultant mitochondrial Ca^2+^ overload would lead to accumulation of mitochondrial superoxide anions, and ultimately facilitate Ca^2+^-induced opening of mPTP (Tikunov et al., 2010). That the closed VDAC channel leads to opening of the mPTP may appear to contradict the notion of an increased pore size due to VDAC1 oligomerization that lead to cytochrome *c* release (Zalk et al., 2005). There is currently no consensus on the role of either the closed state or oligomerized state of VDAC1 in the induction of apoptosis. Both states could lead to apoptosis depending on the triggering stressor.

## Role of VDAC in neoplastic diseases

Cancer cells exhibit a profound change in energy metabolism and ROS production in mitochondria (Cheng et al., 2016; Panieri and Santoro, 2016). Mitochondrial involvement in the etiology of neoplastic diseases has been suggested for some time, since the renowned Nobel prize-winning German physician and scientist, Otto Heinrich Warburg, hypothesized the importance of the organelle in tumorigenesis (Warburg, 1956; Koppenol et al., 2011). Indeed, in recent years, alterations of mitochondrial functions have been implicated as key features in cancer cells and other pathologies, including neurodegenerative diseases, such as Parkinson's and Alzheimer's disease (Wallace, 2012). However, in contrast to neurodegenerative diseases or cardiac diseases, impaired mitochondrial function in tumor cells tends to be the cornerstone for cell survival.

Metabolic dysregulation in cancer has long been regarded as a product of tumorigenesis to support tumor growth and survival. Cancer cells can survive because they are typically characterized by high rates of glycolysis despite an oxygenated cellular environment. This shift to aerobic glycolysis by cancer cells, the “Warburg effect”, is not only a consequence of malignant transformation but is regarded as a crucial hallmark of cancer (Maldonado and Lemasters, 2012; Kruspig et al., 2014). It is postulated that the “Warburg effect” is caused by the closure of VDAC resulting in reduced conductance, which leads to global restriction of the OMM permeability linked to aerobic glycolysis (Lemeshko, 2014a,b).

Although aerobic glycolysis is an inefficient mechanism for ATP production, alterations in glucose metabolism enhance cancer cells' resistance to apoptosis. A pivotal player in the switch from oxidative phosphorylation to aerobic glycolysis may be HKII binding to VDAC for cancer cell survival (Azoulay-Zohar et al., 2004; Shoshan-Barmatz et al., 2009). In fact, the involvement of VDAC1 in cancer metabolism, through its association with HK, has been documented (Shoshan-Barmatz et al., 2009; Leanza et al., 2014). In many types of cancer cells, cytosolic HK-I and HK-II levels are elevated with increased translocation to the OMM. Since HK binds to VDAC1, it has direct access to mitochondrial ATP for phosphorylation of glucose to glucose-6-phosphate (G-6-P), a rate-controlling step in glycolysis. The coupling between VDAC1 and elevated levels of HK leads to a high glycolytic rate resulting in enhanced generation of lactate, a key component in promoting cell growth and protecting against mitochondria-mediated cell death in cancer cells (Abu-Hamad et al., 2008; Maldonado and Lemasters, 2012; Shoshan-Barmatz et al., 2014).

In cancer cells, the level of free tubulin increases to regulate VDAC conductance during the metabolic transformation, with impact on bioenergetics. The ΔΨ_m_, and concomitantly VDAC conductance increases or decreases in tumor cells depending on the amount of free tubulin (Maldonado et al., 2010, 2013; Maldonado and Lemasters, 2014). In addition, tubulin binding to VDAC is dependent on the state of VDAC phosphorylation and regulates OMM permeability to respiratory substrates (Maldonado et al., 2010). Loss of ΔΨ_m_ and reduced VDAC phosphorylation have been associated with high fat mediated hepatic steatosis. This may represent a hallmark for chronic lipid exposure during liver steatosis (Martel et al., 2013) with potential implications for liver cancer (Ohata et al., 2003).

In addition to the aforementioned functional consequences, VDAC1-HK interaction has also been shown to prevent apoptosis. Studies have shown that HK bound to VDAC prevents mitochondria-mediated apoptosis triggered by Bax or Bak (Majewski et al., 2004). Furthermore, it was reported that undocking of HK from mitochondria leads to cytochrome c release. This suggests that HK, when bound to VDAC, blocks the interaction of VDAC with the pro-apoptotic members of the Bcl-2 family of proteins. Mitochondria-bound HK is also found to attenuate ROS induced apoptosis by reducing mitochondrial ROS generation (Da-Silva et al., 2004). Insofar as VDACs are involved in mitochondria ROS production, HK bound to VDACs would promote protection against oxidative stress by minimizing permeabilization of the OMM and subsequent release of cytochrome *c*, an important antioxidant (Mathupala et al., 2006). Consequently, in tumor cells with elevated levels of HK bound to VDAC1, apoptosis is suppressed and proliferation is facilitated. Thus, the disruption of the HK-VDAC1 interaction should facilitate cell death. Single mutations or N-terminus truncation of VDAC that are essential for HK binding have been shown to abrogate HK-induced protection against apoptosis and even potentiate caspase-2-induced mitochondrial damage (Zaid et al., 2005; Abu-Hamad et al., 2008, 2009). The importance of the HK-VDAC1 interaction in cell survival and protection against apoptosis is well documented, and provides an attractive target for cancer therapy and the development of anti-cancer drugs (Galluzzi et al., 2008; Shoshan-Barmatz and Golan, 2012; Krasnov et al., 2013).

Interestingly, silencing VDAC1 expression has been shown to induce inhibition of tumor growth (Arif et al., 2014). Furthermore, preventing Bak sequestration by VDAC2 or inducing Bak activation by dissociation from VDAC2 is considered a plausible approach to trigger apoptosis in tumor cells (Cheng et al., 2003; Lazarou et al., 2010). The pro-apoptotic protein Bcl-Xs-mediated disruption of the VDAC2-Bak association has been reported to be an effective strategy to induce apoptosis in melanoma cells (Cheng et al., 2003; Lazarou et al., 2010; Plötz et al., 2012). It has also been reported that VDAC3 is the most important among the three VDAC isoforms to sustain ΔΨ_m_ in cancer cells, because the high levels of free tubulin inhibit VDAC1 and VDAC2, but not VDAC3 (Maldonado et al., 2013). A shift in this dynamic relationship between the VDACs and tubulin in regulating ΔΨ_m_ has implications for the Warburg effect and subsequently, cancer growth and proliferation (Maldonado et al., 2013; Maldonado and Lemasters, 2014).

In marked contrast to the therapeutic strategy of targeting mitochondria in neurodegenerative and ischemic heart diseases (discussed later), which is prevention of cell death, the main goal of targeting the mitochondria in neoplastic disease, specifically VDAC1, is to kill malignant cells by inducing apoptosis. Pharmacological disruption of the HK-VDAC1 association has been shown to decrease cancer cell survival and facilitate cell death (Simamura et al., 2008; Shoshan-Barmatz and Golan, 2012; Wenner, 2012; Krasnov et al., 2013). For example, clotrimazole, an azole derivative, has potential anti-cancer effects. Clotrimazole has been shown to disrupt HK-VDAC1 association, and recently was reported to inhibit human breast cancer cell proliferation, viability and glycolysis by altering the rates of glucose uptake, mitochondrial activity and ATP generation (Furtado et al., 2012). Another potential anti-cancer drug, 3-bromopyruvic acid (3-BP), is an alkylating agent that is effective in inhibiting glycolysis in aggressive liver tumor cells by disrupting the HK-VDAC interaction (Gong et al., 2014), and inhibiting proliferation in a human breast cancer MCF-7 cell line by down-regulation of Bcl-2 (Liu et al., 2009; Kwiatkowska et al., 2016). In an animal model, 3-BP eradicated advanced stage, positron emission tomography (PET) positive hepatocellular carcinomas without apparent harm to the animals (Ko et al., 2004). Recently 3-BP has also been shown to be an activator of oxidative stress by depleting antioxidants and inactivating antioxidant enzymes (Kwiatkowska et al., 2016). Another promising cancer fighting agent, jasmonate, derived from a plant stress hormone, has also been shown to act directly on mitochondria of cancer cells by detaching HK from the OMM and inducing cell death via mPTP opening (Rotem et al., 2005).

Other potential cancer-fighting agents also appear to target VDAC1 directly to alter its channel's gating properties. One such group of drugs is the avicins, a family of triterpenoid saponins. Studies have reported on the ability of avicins to reduce VDAC1 channel conductance with a subsequent decrease in cell energy metabolism and triggering of the apoptotic pathway by permeabilization of the OMM (Haridas et al., 2007). Another potential candidate for cancer therapy is oblimersen, an 18-mer phosphorothioate anti-sense oligonucleotide (also known as G3139) (Lai et al., 2006). Oblimersen targets VDAC and results in a significant reduction in channel conductance and decrease in metabolic fluxes across the OMM. This perturbation in the OMM is thought to induce the formation of a protein-conductive pathway that increase permeabilization of mitochondrial membranes and allow the release of cytochrome *c* to initiate the intrinsic apoptotic process. In fact, a reduction in VDAC channel activity or increased membrane permeabilization by a chemotherapeutic agent that is currently in widespread clinical use, cisplatin (*cis*-diamminedichloroplatinum II), is available for the treatment of head and neck cancer and other types of malignant cancer (Yang et al., 2006). This study demonstrated VDAC1 as effector of mitochondrial membrane permeabilization via activation of Bax. In a recent review, Shoshan-Barmatz et al. (2014) highlighted therapeutic strategies involving the use of siRNA to impair energy and metabolic homeostasis leading to the arrest of cancer cell growth and proliferation, as well as the use of VDAC1-based peptides that interact with anti-apoptotic proteins to induce apoptosis. Thus, efficient exploration of targeted drugs and genetic approaches that act on mitochondrial VDAC1 to antagonize tumor growth and proliferation is a promising strategy to treat cancer.

## Role of VDAC in neurodegenerative diseases

Mitochondrial dysfunction also plays a key role in neurodegenerative diseases. As an excitable tissue, the central nervous system is particularly sensitive to oxidative stress. Numerous neurological diseases, for example Parkinson's disease, multiple sclerosis, Huntington's disease, and Alzheimer's disease (AD) involve mitochondrial dysfunction. (For an extensive review, see Camara et al., 2010). In this section, we will focus on AD as an example where the role of mitochondrial VDAC1 has been implicated in the etiology and progression of the disease (Manczak and Reddy, 2012).

AD is a destructive neurodegenerative disease and the most common form of dementia (Selkoe, 2001). It is characterized by accumulation of amyloid plaques in brain tissue and progressive neuroanatomical and cognitive impairment with advanced age. Although the underlying mechanism of the disease is not well understood, oxidative stress and mitochondrial dysfunction have been implicated in the development and progression of AD. One of the major pathological hallmarks of the disease is the accumulation of amyloid beta (Aβ) in the extracellular space. However, studies have reported on the accumulation of Aβ in subcellular organelles, including mitochondria (Hansson Petersen et al., 2008). Evidence shows that Aβ is taken up into mitochondria via the outer membrane transport protein, translocase of the outer membrane (TOM), and accumulate in the IMM and cristae, likely via translocase of the inner membrane (TIM), where they could interfere with electron transfer and increase ROS production (Hansson Petersen et al., 2008; Pinho et al., 2014).

Recent evidence has shown direct interaction of Aβ with VDAC1 (Manczak and Reddy, 2012; Smilansky et al., 2015). This interaction occurs for both the putative plasma membrane (discussed below) VDAC1 and mitochondrial VDAC1. It is worth noting that Aβ interaction with plasma membrane VDAC facilitates penetration of Aβ into the cell (Thinnes, 2015a,b). At the mitochondrial level, Aβ interaction with VDAC1 results in the detachment of the anti-apoptotic protein, HK, an increase in channel conductance likely by inducing VDAC1 oligomerization, and cytochrome *c* release (Reddy, 2013a,b). Incidentally, increased mitochondrial VDAC1 expressions levels have been shown to correlate with the progression of AD, and reduced VDAC1 expression, such as that observed in VDAC1^+/−^ mice protected against AD-related toxicities (Manczak and Reddy, 2013; Manczak et al., 2013). The increased VDAC1 expression in AD is accompanied by decreased ATPase (complex V) activity (Manczak and Reddy, 2013; Manczak et al., 2013). In the VDAC1^+/−^ mice, it is reported that ROS production and lipid peroxidation levels are reduced, while cytochrome oxidase (complex IV) activity and ATP levels are elevated, indicative of enhanced mitochondrial function (Manczak et al., 2013).

Interestingly, studies have also shown that the interaction of VDAC1 with Aβ can lead to channel closure. In particular, VDAC1 is found to interact with phosphorylated tau, another key component in AD pathogenesis, and together with Aβ, lead to channel block (Manczak and Reddy, 2012). This combination between tau and Aβ leads to derangements in metabolite fluxes across the OMM that results in defective oxidative phosphorylation. Whether Aβ enhances or diminishes VDAC1 conductance appears to be time-dependent: incubation with Aβ leads to greater conductance while a direct effect appears to block the channel pore. This effect on channel function adds to the complexity and difficulty in delineating the molecular mechanisms underlying AD. Nevertheless, these studies highlight the role of VDAC1 dysfunction in the etiology of AD. Down-regulation of VDAC1 and/or preventing its interaction with Aβ and phosphorylated tau could potentially preserve mitochondrial function, slow the progression of AD, and ultimately improve cognitive function in patients (Cuadrado-Tejedor et al., 2011; Manczak and Reddy, 2012).

In addition to its interaction with Aβ and phosphorylated tau, VDAC1 is also found to undergo post-translational modification (PTM) because of oxidative stress, another key pathogenic factor in the development of AD. In a study identifying nitrated proteins in AD patients using proteomics approach, a significant increase in protein nitration of VDAC1 was detected in hippocampal samples (Sultana et al., 2006). Nitration, triggered by increased levels of the potent reactive nitrogen species (RNS) peroxynitrite (ONOO^−^), a product of the reaction between nitric oxide (NO^•^) and superoxide anion (${\text{O}}_{2}^{\cdot -}$), can irreversibly damage proteins, and lead to altered functional characteristics of the affected proteins. Nitration of VDAC1 could result in modified channel function that ultimately impairs cognitive function. Deleterious consequences of nitration-induced changes in VDAC conductance have been reported in cardiac mitochondria (Yang et al., 2012) (see section “Role of VDAC in Cardiac Injury” below). Recent studies reported on other types of PTMs of VDAC1 in AD. For example, VDAC1 was reported to be significantly carbonylated by acrolein in AD patients (Mello et al., 2007). Acrolein, the most reactive of the unsaturated aldehydes, is formed through Fe-catalyzed oxidation of arachidonic and docosahexaenoic acids, and is known to increase protein carbonylation (Esterbauer et al., 1991; Uchida et al., 1998). In proteomics studies, acrolein was reported to be significantly elevated in the brain of AD patients (Lovell et al., 2001). Hence, oxidative damage in the AD brain, including nitration and carbonylation of VDAC1, likely impairs channel function and contributes to the pathogenesis and progression of AD, with concomitant cognitive impairment. This notion is further espoused by the observations that VDAC1 deficient transgenic mice exhibit deficits in long-term potentiation and learning behavior (Weeber et al., 2002).

Like PTMs of VDAC1, significant S-nitrosylation of VDAC2 has also been identified in distinct regions of the brain from AD patients. NO^•^ triggered S-nitrosylation of VDAC2 could potentially alter protein function and lead to dysregulation of Ca^2+^ dynamics in mitochondria (Zahid et al., 2014). Although the functional impact of S-nitrosylation of VDAC2 has not been established, its impact on VDAC1 has been reported. Exogenous NO^•^ was shown to display a biphasic effect on VDAC1 isolated from cardiac mitochondria, i.e., decreasing channel conductance at lower concentrations while increasing conductance at high concentrations (Cheng et al., 2011). Other modifications of VDAC may contribute to the development and progression of AD. It has been reported (Fernandez-Echevarria et al., 2014) that Aβ exposure enhanced the dephosphorylation of VDAC1 that correlated with cell death, which was reversed in the presence of tyrosine phosphatase inhibitors.

These results demonstrate that Aβ is also involved in alterations of the phosphorylation state of VDAC in neurons of AD. Recent studies also revealed that glycogen synthase-3β (GSK-3β) is elevated in AD-affected tissues, and is critically involved in dissociating VDAC1 from HK. This would disrupt glucose metabolism, promote mitochondrial dysfunction and activate apoptotic cell death (Smilansky et al., 2015).

AD is postulated to be more prevalent in elderly females than their male counterparts, and this sexual dimorphism is attributed, in part, to the declining levels of estrogen and the role of estrogen receptors (Lan et al., 2015). It is also suggested that VDAC-estrogen receptor interaction may be important for maintaining channel inactivation and contributing to neuronal preservation against Aβ injury (Lan et al., 2015). Thus, modulation of the channel via its interaction with estrogen receptors may contribute to the development and progression of AD pathology. Consequently, it is worth noting that unraveling the underlying mechanisms of the targeted PTM of VDAC or its interaction with other proteins could be a harbinger for potential novel approaches to assuage or prevent the onset or progression of AD, especially if the mitochondrial markers/targets for the disease can be identified.

## Role of VDAC in cardiac injury

Ischemic heart disease (IHD) has become the leading cause of death worldwide. In IHD, stoppage of flow through the coronary vessels causes irreversible cell death. As the “powerhouse” of the cell and with their large number in cardiomyocytes, mitochondria play critical roles in cell dysfunction during cardiac ischemia and reperfusion (IR) injury. During the ischemic insult, mitochondria are the main source of ROS generation in cardiomyocytes and as such have become prominent in pharmacological intervention or genetic manipulations in mitigating cell damage and death. Thus, mitochondrial abnormality has been recognized as a hallmark of cardiovascular diseases, including IHD (Camara et al., 2010, 2011). To date, many of the studies have pointed to damages of mitochondrial proteins as main contributing factors in mitochondria-mediated cell demise. Some of the major contributing factors in cellular damage during cardiac IR injury are damages to the ETC complexes (Aldakkak et al., 2008a; Chen et al., 2010; Gadicherla et al., 2012; Xu et al., 2014; Yang et al., 2014), and the inability of mitochondria to maintain normal resting ΔΨ_m_, which leads to impaired oxidative phosphorylation needed to generate ATP for cellular function. Ischemia leads to diminished ATP content/production, resulting in energy stress and in a vicious cycle that leads to excess ROS production and further oxidative stress. In this case, lack of ATP leads to further mitochondrial functional derangement, loss of cellular cationic homeostasis and subsequent cell death by necrosis. As a matter of fact, during IR, mitochondrial derangement could lead to membrane permeabilization, i.e., mPTP opening or VDAC-mediated OMM permeabilization, which leads to the release of cytochrome *c* and other apoptotic factors that contribute to apoptosis.

The magnitude of myocardial injury and potential for reduced tissue infarction following IR is related to the duration of ischemia and successful reperfusion (Riess et al., 2004). A prolonged cardiac ischemia and subsequent reperfusion inflicts significant damage to mitochondria that results in cellular energy deprivation and irreversible cardiac injury. We have observed (Camara and Stowe, unpublished) that as ischemia time increases in the *ex vivo* perfused heart, ROS production increased proportionally, when measured continuously with a fiber optic probe placed against the left ventricular free wall (Stowe et al., 2006; Camara et al., 2007; Aldakkak et al., 2011). After 50 min or more of ischemia, ROS generation, paradoxically, declined, which may be attributed to the loss of viable mitochondria; this could lead to irreversible cardiomyocyte damage. Indeed, studies show that it is the ischemic time rather than reperfusion that determines the magnitude of cytochrome *c* release during cardiac IR (Lesnefsky et al., 2004). Loss of cytochrome *c* as noted above, diminishes the scavenging capacity of mitochondria (Camara et al., 2010) and this, in a positive feedback manner, leads to more ROS production and further damage to mitochondria. The activities of the ETC complexes are also impacted by the length of ischemia, with complex I activity decreasing within the first 10–20 min of ischemia (Rouslin and Ranganathan, 1983). We reported that targeting the ETC (complex I), which is the primary source of ROS production during cardiac IR, attenuates mitochondrial ROS production and preserve cardiac function (Aldakkak et al., 2008a). Furthermore, a recent study showed that the modulation of complex I activity by S-nitrosation is cardioprotective against IR injury (Chouchani et al., 2013). Other events that occur during IR include oxidation of mitochondrial redox state (oxidized NADH/FAD), increased mitochondrial free Ca^2+^, decreased mitochondrial membrane potential (ΔΨ_m_) and increased cytochrome *c* release, which cumulatively lead to reduction in ATP production (Aldakkak et al., 2011). Hence, targeting ROS and mitochondrial Ca^2+^ using mitochondria-targeted ROS scavengers, modulators of the ETC (Aldakkak et al., 2008a; Yang et al., 2014), or mitochondrial specific inhibitors of Na^+^/H^+^ exchangers or Na^+^/Ca^2+^ exchangers have all been reported to reverse mitochondrial damage and protect the heart (An et al., 2006; Aldakkak et al., 2008b).

Although the precise role of VDAC in cardiac IR injury has not been delineated, several lines of evidence show that it plays a pivotal role in IR injury and cardioprotection. Modulation of VDAC by cytosolic proteins has been shown to either induce cell death or prevent it during IR injury. As noted above, HK interaction with VDAC confers protection for tumor cells against apoptosis. Similar strategies have been postulated for several cardioprotective strategies, for example, in ischemic preconditioning (IPC) and post-conditioning (POC) (Camara et al., 2011). In these strategies, increased association between HK and mitochondrial VDAC has been shown to reduce cell death in cardiomyocytes. HK has a hydrophobic N-terminal sequence that is essential for its docking with mitochondrial VDAC (Azoulay-Zohar et al., 2004). This docking promotes VDAC closed state and reduces permeability to ATP/ADP transfer across the OMM. Under de-energized state, it has been reported that reduced transport of ATP/ADP across the OMM by inhibiting VDAC opening significantly attenuates myocardial IR injury (Steenbergen et al., 2009). ROS emission and infarct size after reperfusion parallel the degree of HK dissociation from mitochondria (Pasdois et al., 2013). Cancer cells with increased HK-VDAC association are thought to be less susceptible to IR damage compared to normal cells.

Studies in cardiac mitochondria demonstrated an interaction of PKC-ε, which translocates to mitochondria, with VDAC1 in IPC and POC cardioprotection (Baines et al., 2003; Korzick et al., 2007). IPC and POC strategies also protected the heart against IR injury in a hypertensive animal model; the cardioprotection was correlated with increased association of phospho-PKC-ε with VDAC (although the isoform was not specified). Alternatively, HK detachment from VDAC by pro-apoptotic proteins (Bak and Bax) or by GSK-3β during IR has been linked to OMM permeabilization and cell death (Majewski et al., 2004; Chiara et al., 2008). In the heart, GSK-3β has several important roles. GSK-3β phosphorylates VDAC, which is modulated by Akt (protein kinase B). Decreased phosphorylation of VDAC, which is induced by inhibition of GSK-3β, can reduce ATP entry into mitochondria (Das et al., 2008). Thus, permeabilization of OMM by GSK-3β is suppressed by activation of Akt, which phosphorylates GSK-3β at Ser9, resulting in dislodging of the protein from its binding site and mitigating cell damage (Majewski et al., 2004). Other studies have shown phospho-serine-GSK-3β-mediated cytoprotection is achieved by increased threshold for mPTP opening, possibly through phosphorylation of VDAC (Pastorino et al., 2005; Nishihara et al., 2007). Additional evidence shows that Akt also is cytoprotective by phosphorylating HK and concomitantly enhancing its binding to mitochondria (Miyamoto et al., 2008; Roberts et al., 2013), which prevents GSK-3β binding to VDAC (Das et al., 2008; Martel et al., 2014).

The close proximity of VDAC1 and SR Ca^2+^ handling proteins [e.g., inositol 1,4,5-triphosphate receptors (IP3R)] in the MAM domain provide direct Ca^2+^ signal from SR to mitochondria (Patergnani et al., 2011). This link has been shown to be significant during IR when there is increased translocation of GSK-3β at the MAM domain (Gomez et al., 2016). It may increase Ca^2+^ leak from SR and contribute to increase mitochondrial Ca^2+^ overload via modified VDAC. A recent study showed that pharmacological and siRNA inhibition of GSK-3β at reoxygenation after hypoxia reduced Ca^2+^ leak from SR in cardiomyocytes, limited both cytosolic and mitochondrial Ca^2+^ overload and reduced cell death (Gomez et al., 2016). It is worth noting that the pro-apoptotic Bcl-2 family of proteins is also localized in the MAM region where they influence mitochondrial permeability and mitochondrial functional integrity, presumably via VDAC, during Ca^2+^ overload or oxidative stress (Giorgi et al., 2015). HK-VDAC association is known to prevent apoptosis by interfering with Bax binding to mitochondria and impeding VDAC1 oligomerization (Keinan et al., 2010; Shoshan-Barmatz et al., 2010) (see below for more details).

Recent studies have examined how excess ROS and RNS exert their deleterious effects via PTMs of key mitochondrial proteins, resulting in mitochondrial dysfunction. Several studies have reported on the involvement of PTMs of VDAC in IR injury and in cardioprotection. Foremost in these novel findings is that PTM of VDAC by NO^•^ and ONOO^−^ may be important in regulating the function of the channel, thereby preserving mitochondrial function and cell survival, or contributing to mitochondrial dysfunction and cell death. Nitration of amino acid residues in the ETC complexes and other proteins (e.g., VDAC, ANT) likely leads to altered protein function (Zhang et al., 2010; Yang et al., 2012). We reported that NO^•^ caused a concentration-dependent biphasic inhibition of the cardiac VDAC incorporated in a planer lipid bilayer (Cheng et al., 2011). The implications of these findings is that PTM of VDAC by NO^•^ and other N_2_ containing molecules may be important in regulating the function of the channel and thereby mitochondrial function and cell fate. As mentioned above, we reported for the first time that nitration of VDAC contributes to the pathology associated with cardiac IR injury (Yang et al., 2012). VDAC nitration of specific tyrosine residues was associated with increased ${\text{O}}_{2}^{\cdot -}$ and ONOO^−^ production, which significantly compromised cardiac function on reperfusion. Treatment with resveratrol, a scavenger of ROS and RNS, or NG-nitro-L-arginine methyl ester (L-NAME), a NOS inhibitor, reduced *ex vivo* VDAC nitration, reduced ${\text{O}}_{2}^{\cdot -}$ and ONOO^−^ generation, and improved cardiac function on reperfusion. In the same study, we also reported that VDAC nitration resulted in VDAC oligomerization, which could have contributed to the uncharacteristically large conductance of the channel when incorporated into lipid bilayers.

Phosphorylation of VDAC is an important PTM with strong implications for beneficial or injurious effect during IR. Whether phosphorylation or dephosphorylation of VDAC is beneficial to cardiac function during IR has not been resolved. For example, the cardioprotective effects of PD169316, an inhibitor of the p38 mitogen-activated protein kinase, resulted in a significant reduction in ischemia-induced phosphorylation of VDAC1, specifically a reduction in tyrosine phosphorylation (Schwertz et al., 2007). The functional consequence of tyrosine phosphorylation of VDAC1 was not established in that study. Nevertheless, the study demonstrated that reduced tyrosine phosphorylation of VDAC1 may underlie a mechanism of cardioprotection. For instance, inhibition of GSK-3β was reported to reduce IR injury; this involved a reduction in phosphorylation of VDAC, specifically VDAC2 (Das et al., 2008). Endostatin-induced apoptosis in endothelial cells were found to involve the phosphorylation of VDAC1 (Yuan et al., 2008). On the other hand, phosphorylation of VDAC has also been implicated as a cardioprotective mechanism. A significant increase in *O*-linked β-*N*-acetylglucosamine (*O*-GlcNAc) levels has been reported in IPC, and the pharmacological augmentation of *O*-GlcNAc has resulted in a significant decrease in infarct size following ischemia (Jones et al., 2008). *O*-GlcNAc induced modification of VDAC, analogous to protein phosphorylation, was detected in that study, suggestive of a cardioprotective PTM. In endothelin-1 induced cardiac hypertrophy, VDAC phosphorylation was reduced, an effect that was mimicked by a GSK-3β inhibitor. The reduced phosphorylation was associated with mitochondrial membrane depolarization (Javadov et al., 2009b).

Identifying PTMs of VDAC and establishing the resultant functional changes should help define its potential pathogenic role in disease states and to provide information on how and where to target therapies to mitigate cell death. In this scenario, targeted protein modifications may be a unique therapeutic approach for the rescue of mitochondrial proteins and for protection against cardiac oxidative (${\text{O}}_{2}^{\cdot -}$) and/or nitrosative (NO^•^/ONOO^−^) damage due to I/R injury.

## Structural impact of site-specific PTM and functional consequence of VDAC1

It was less than a decade ago when the 3-dimensional structure of the VDAC1 isoform was determined (Bayrhuber et al., 2008; Hiller et al., 2008; Ujwal et al., 2008; Figure 2A). The availability of the VDAC1 structure allows for investigation of the intricate molecular details of the protein's structure-function relationship, most importantly the structural basis for conductance, ion selectivity and gating of this ion channel. The channel's permeability to small molecules, ions and nucleotides has been investigated in several studies employing molecular dynamics (MD) or Brownian dynamics (BD) simulation (for a comprehensive review, see Noskov et al., 2016).

The conductance and ion selectivity captured by the simulations are in general consistent with experimentally measured values. Based on these calculations, an open (high-conductance) state can be assigned to the conformations captured in NMR spectroscopy and crystal structures. The closure of the pore (mechanism of gating), however, has not been structurally well characterized. A mutagenesis study with MD simulations suggested that the pore closure might be a result of geometrical distortion of the barrel, i.e., the pore is elliptically “squeezed” under gating conditions (Zachariae et al., 2012).

In terms of substrate selectivity, it has been predicted that a specific ATP binding site might exist within the VDAC pore (Figure 2B) due to a largely reduced in-pore diffusion constant calculated for this substrate compared to its bulk value (Rostovtseva and Bezrukov, 1998). Free energy analysis employing non-equilibrium MD trajectories of ATP passage across the channel pore has identified significant contributions from the N-terminal helical region to this phenomenon (Noskov et al., 2013). This is further confirmed by the discovery of an ATP-bound crystal structure of murine VDAC1, which demonstrates direct engagement of the phosphate moieties of ATP in salt-bridges with the N-terminal region (Choudhary et al., 2014; Figure 2C). Utilizing hundreds of short equilibrium MD simulations, nucleotide permeation pathways have been reconstructed in a Markov state model (Choudhary et al., 2014). Notably the highest ranked pathway identified in this study is similar to that identified in a separate study using MD simulations on a much smaller scale (Krammer et al., 2015).

Since the interior of VDAC1 is highly polar with multiple salt bridges involved in the barrel structure and lining the substrate permeation pathway, perturbation to the electrostatic charge distributions within the channel is expected to largely alter its electrophysiological properties, and thus the channel's conductance. A study with MD and BD simulations on several VDAC1 mutants whose charged residues were modified showed that mutations in the interior of the pore do affect ion selectivity, whereas mutations on the rim of the barrel have little effect (Krammer et al., 2013). Furthermore, lowering pH has been demonstrated to promote voltage-induced closure (Teijido et al., 2014). However, due to the intrinsically asymmetric distribution of charged residues, whether the pH change is originated from the cytosol or the IMS will result in different open probabilities and averaged conductance (Teijido et al., 2014). Many PTMs to VDAC, particularly phosphorylation, introduce additional negative charges to the barrel and change the charge distribution inside the pore (Figure 2D). While many of these PTMs have profound physiological effects (Martel et al., 2014), whether a specific PTM changes the conductance, selectivity or voltage dependence, or even the interaction of the protein with other regulatory mechanisms would require further studies at the molecular level.

Intriguingly, in the VDAC structures (Figure 2), a charged residue Glu73 is located at the mid-point of the barrel with its side chain facing away from the pore and toward the lipids in the center of the membrane. Neutralizing this residue in MD simulations greatly reduced the structural fluctuations (Villinger et al., 2010), which is further evidenced by the highly ordered E73V mutant structure recently resolved with NMR (Jaremko et al., 2016). It is known that Glu73 forms a Ca^2+^ binding site (Israelson et al., 2007), while HK-I binding of VDAC1 is abolished by E73Q mutation (Zaid et al., 2005). How these interactions and effects at a location right in the middle of a lipid bilayer can be of high impact to the fate of the cell may become a hot topic of research.

## Does VDAC exist in the plasma membrane?

The response to this question seems to depend largely on the tissue type. Unlike the OMM VDAC, there is currently no consensus that VDACs are universally present in the sarcolemma of eukaryotic cells. The notion of VDAC in plasma membrane was first reported in 1989, after the revelation by Thinnes lab that porin exists in human B-lymphocyte plasma membrane (Kayser et al., 1989; Thinnes et al., 1989). This observation was subsequently supported by other studies from other cell types, including post-synaptic membrane fractions from the brain and in caveolae of neurons (Moon et al., 1999). In subsequent studies, an array of approaches was used to ascertain the purity of the protein in the plasma membrane of these cells (Schindler et al., 2006). However, other studies have disputed the existence of VDAC in the plasma membrane and have discounted previous findings as artifacts or contaminations (Yu et al., 1995). In that study, the authors suggested that extra-mitochondrial localization of VDAC were likely due to unspecific immunoreactions and redistribution in subcellular compartments during procedures involving the use of detergent. It is worth noting that there is no evidence to date that unambiguously demonstrate the presence of the protein in cardiomyocyte plasma membrane. Nonetheless, in those cells where the channel has been identified in the plasma membrane, some functional roles have been suggested (De Pinto et al., 2010b). As alluded above (Role of VDAC in Neurodegenerative Disease), plasma membrane VDAC has also been implicated in the transport of Aβ into the cell (Thinnes, 2015b). The study showed that silencing VDAC1 expression by siRNA prevented Aβ entry into the cytosol and prevented cytotoxicity. In other cells, for example, in fibroblasts and epithelial cells, plasma membrane VDAC1 may have physiological relevance in regulating extracellular ATP release. It has been reported (Okada et al., 2004) that plasma membrane VDAC1 independently regulated ATP release and ATP-mediated cell volume responses to hypertonicity. Others have suggested that plasma membrane VDAC1 can function as a redox enzyme reductase, a function not clearly attributed to VDAC1 in mitochondria (Baker et al., 2004). This role of VDAC is postulated to be involved in the maintenance of cellular redox homeostasis. Since this study was conducted in Namalwa cells, special human lymphoma cell lines, the extrapolation of the observations to other cells may be limited. Lastly, in normal neuronal cells, it has been reported that plasma membrane VDAC plays a role in the early stages of neuronal apoptosis, because anti-VDAC antibodies block the apoptotic process (Elinder et al., 2005), and in the development of osteoclasts (bone resorption cells) during bone remodeling (Kotake et al., 2013).

The existence of plasma membrane VDAC and its physiological significance is not as well delineated as its mitochondrial counterpart. Furthermore, in those cells where VDAC has been identified in the plasma membrane, how or whether it has a functional crosstalk with mitochondrial VDAC under normal and pathological conditions (e.g., oxidative/nitrosative stress, mitochondrial Ca^2+^ overload) remains to be explored. The fact that possible artifacts from mitochondrial membrane during plasma membrane VDAC isolation procedures could occur, a thoughtful consideration on a *bona fide* plasma membrane VDAC requires further scrutiny.

## Perspective

After decades of intense investigation, VDAC has evolved from a mere porin to a channel at the crossroad between metabolism, cell survival pathways and cell death. VDAC is now recognized not just as a conduit for the transport of metabolites/ions and regulator of metabolic and energetic function, it is also a convergence point for a variety of survival and cell death signaling pathways. In this review, we examined the role of VDACs, particularly VDAC1 in mitochondria, in the physiology and pathophysiology of neoplastic and neurodegenerative diseases and cardiac IR injury. This review is narrow in its focus, but this is not reflective of the extent the subject is covered in the literature. There remains a lot of grounds to cover and uncover on the physiological role of VDAC in mitochondrial function and dysfunction. Clearly, VDAC is critical in the docking of both cytosolic and mitochondrial proteins, and its physiological and pathophysiological roles are mediated in part by various PTMs. A better appreciation of these PTMs on protein structure and function and the impact this has on the transformation from a pore to a receptor for other proteins, or from a mediator of cytoprotection to a mediator of cell death, is important for unraveling the implications of the protein as a potential therapeutic target. In this context, VDAC can be recognized as a gatekeeper for normal mitochondrial function and a crucial factor in both cytoprotection, as in cancer cells, and a mediator of mitochondria-induced apoptosis, as in IR injury and AD. As such, VDAC is a highly enigmatic protein whose dysfunction can contribute to the pathogenesis of multitude of diseases.

## Author contributions

AC, YZ, PW, ET, and WK all contributed to the writing and editing of the manuscript.

### Conflict of interest statement

The authors declare that the research was conducted in the absence of any commercial or financial relationships that could be construed as a potential conflict of interest.

## Abbreviations

Aβ: amyloid β
AD: Alzheimer's disease
ANT: adenine nucleotide translocase
BD: Brownian dynamics
ETC: electron transport chain
G-6-P: glucose-6-phosphate
GSK-3β: glycogen synthase kinase-3 beta
HK: hexokinase
HVDAC: human voltage dependent anion channel
IMM: inner mitochondrial membrane
IHD: ischemic heart disease
IP3R: inositol trisphosphate receptor
IMS: inter-membrane space
IPC: ischemic preconditioning
IR: ischemia and reperfusion
L-NAME: L-NG-Nitroarginine methyl ester
LTP: long-term potentiation
MAM: mitochondria associated SR membrane
mCK: mitochondrial creatine kinase
MD: molecular dynamics
mPTP: mitochondrial permeability transition pore
NADH: nicotinamide adenine dinucleotide (reduced)
NO^•^: nitric oxide radical; O-linked N-acetylglucosamine (O-GlcNAc)
OMM: outer mitochondrial membrane
ONOO^−^: peroxynitrite
${\text{O}}_{2}^{\cdot -}$: superoxide anion radical
PET: positron emission tomography
POC: post-conditioning
PTM: posttranslational modification
RNS: reactive nitrogen species
ROS: reactive oxygen species
siRNA: small interfering RNA
SR: sarcoplasmic reticulum
TCA: tricarboxylic acid
TOM and TIM: outer and inner membrane translocases, respectively
TSPO: translocator protein
VDAC: voltage dependent anion channel
ΔΨ_m_: mitochondrial trans-membrane potential.
